# Molecular Survey and Genetic Characteristics of Vector-Borne Pathogens in Domestic Dogs from Four Regions of China

**DOI:** 10.3390/ani13111867

**Published:** 2023-06-03

**Authors:** Fangyuan Yin, Chuanjiang Guo, Dong Li, Zhuojia Tian, Facai Li

**Affiliations:** College of Veterinary Medicine, Southwest University, Chongqing 400715, China

**Keywords:** vector-borne pathogens, *Hepatozoon canis*, pet dogs, 18S rRNA, China

## Abstract

**Simple Summary:**

Canine vector-borne diseases (CVBDs) can affect the health of domestic and wild animals, and their prevalence is increasing worldwide. As potential reservoirs of zoonotic pathogens, dogs might transfer these pathogens to humans. There is limited knowledge about the vector-borne pathogens circulating in dogs in China. To investigate the current epidemiological status and genetic characteristics of *Ehrlichia* spp., *Hepatozoon* spp., and *Mycoplasma* spp., blood samples were collected from healthy pet dogs in four regions of China. There was no evidence of *Ehrlichia* spp. or *Mycoplasma* spp., and only *Hepatozoon canis* was detected in these dogs. High haplotype diversity and the occurrence of genetic variation were observed among these *H. canis* isolates. These results will be useful for developing effective control approaches against CVBDs in companion animals.

**Abstract:**

Canine vector-borne diseases are widely distributed around the world. They are transmitted by arthropods, and many seriously threaten the health of animals and humans. In China, our knowledge of *Ehrlichia*, *Hepatozoon*, and *Mycoplasma* species circulating in dogs is still poorly understood. Therefore, the aim of this study was to understand the prevalence and genetic characteristics of canine *Ehrlichia* spp., *Hepatozoon* spp., and *Mycoplasma* spp. in Chongqing (southwest), Fujian (southeast), Shandong (southeast), and Hubei (central) Provinces of China. Blood samples from healthy pet dogs were processed to detect *Ehrlichia*, *Hepatozoon*, and *Mycoplasma* DNA with PCR. Haplotype and phylogenetic analyses were performed on 18S rRNA sequences. Among 306 dogs, no *Ehrlichia* spp. or *Mycoplasma* spp. were detected, whereas one *Hepatozoon* sp. was detected in 10 (3.27%) of the animals. Only *Hepatozoon canis* was identified and was endemic to Chongqing (2.46%) and Hubei (8.77%). A haplotype analysis identified eight haplotypes among the *H*. *canis* isolates. A phylogenetic analysis showed that the *H. canis* isolates in this study clustered into four clades, together with isolates from different countries and hosts, forming a large group that was clearly separate from other *Hepatozoon* species. These findings provided new information on the epidemiological characteristics of canine vector-borne diseases in China and will be helpful in the development of efficient measures to safeguard the health and well-being of companion animals and their owners.

## 1. Introduction

Canine vector-borne diseases (CVBDs) pose a risk to the health of both domestic and wild animals, and many CVBDs are zoonotic [1]. The pathogens involved in CVBDs include viruses, bacteria, protozoans, and helminths, which are transmitted by hematophagous arthropods such as ticks, mosquitoes, fleas, and lice [2]. CVBDs are widely distributed in tropical, subtropical, and temperate areas, which are suitable for the survival of these vectors [3,4,5]. In recent years, increasing numbers of families have kept pets, and contact with animals is common, increasing the opportunities for the transmission of zoonotic infectious diseases to humans [6]. Therefore, monitoring CVBD infections is important for understanding the health status of pets and their owners and for improving pet welfare.

Hepatozoonosis is a tick-borne disease caused by *Hepatozoon americanum* and *Hepatozoon canis*, which are mainly transmitted by *Amblyomma maculatum* and *Rhipicephalus sanguineus*, respectively [7,8]. Other tick species have been identified as vectors or suspected vectors of *H. canis,* including *A. ovale*, *Dermacentor marginatus*, *Haemaphysalis* (*Hae*) *flava*, *Hae. longicornis*, and *R. turanicus* [9,10,11,12]. The life cycle of these two *Hepatozoon* species is heteroxenous in that they undergo a sporogonic stage in the definitive invertebrate host (e.g., mite, mosquito, or tick), and merogonic and gamontogonic stages in the intermediate vertebrate host [13]. In contrast to other tick-borne pathogens that are transmitted through the tick salivary glands, the transmission of *Hepatozoon* occurs through the ingestion of ticks containing mature oocysts [14]. Other routes by which *Hepatozoon* is transmitted to dogs have been confirmed, including transplacental infections and predation on infected animals [15,16]. *Hepatozoon americanum* is mainly endemic in the United States, and its infection causes fever, leukocytosis, musculoskeletal pain, and often fatal disease [13]. *Hepatozoon canis* is widely distributed and has been described in dogs in Asia, Africa, America, and Europe [17,18,19,20]. Its infections cause lethargy and anemia, but most cases have subclinical symptoms [21]. Wild canids are possible reservoirs of *H. canis* and usually display no clinical signs [22]. *H. canis* is a common parasite in red foxes in Poland (45.7%), Portugal (75.6%), and Spain (91%) [22,23,24]. In Italy, the prevalence of *H. canis* in red foxes (13.4%) is higher than in dogs (3.6%) [25,26], and similar rates have been observed in foxes (27.9%) and dogs (1.8%) in Germany [27]. *H. canis* has also been detected in golden jackals in Israel and Hungary [28,29]. In Israel, the prevalence of *H. canis* in golden jackals (46%) and red foxes (43%) is similar to that in dogs (33.1%) [28,30]. In China, there is little information on *H. canis* infections in wild canids, but they have been sporadically reported in dogs, such as in Beijing, Henan, Jiangsu, Shaanxi, and Xinjiang [31,32]. 

Canine monocytic ehrlichiosis (CME) is another tick-borne disease caused by *Ehrlichia canis*, a gram-negative bacterium of the family Anaplasmataceae. *E. canis* is transmitted by *R. sanguineus* and is mainly distributed in tropical, subtropical, and Mediterranean climates [33,34,35]. *E. canis* is inoculated into the host by ticks through their salivary glands during blood meals. This pathogen can be transmitted by a tick within 3 h of its attachment to a host. *E. canis* primarily infects monocytes in dogs, and its infection may cause thrombocytopenia. After an incubation period of 1–3 weeks, CME has three phases: acute, subclinical, and chronic [36]. In the acute phase, dogs are characterized by fever, anorexia, lethargy, lymphadenomegaly, and splenomegaly. Dogs are likely to be infested with ticks during this phase. In the subclinical or chronic phase, the dog seems to be healthy but becomes a pathogen carrier. Ophthalmic lesions are common and often include anterior uveitis, papilledema, chorioretinitis, and retinal hemorrhage [37]. *E. canis* occasionally infects humans and has been confirmed as causing human disease in Venezuela [38]. All dog breeds are susceptible to CME. However, German Shepherds are more likely to have severe clinical symptoms and a poor prognosis [39]. In clinical cases, detecting *E. canis* morula is rarely used in a blood smear because it occurs at a low incidence (4–6%). Usually, *E. canis* can be diagnosed with serological (e.g., IFAT or ELISA) or molecular (e.g., PCR) techniques. In Europe, *E. canis* has been detected in dogs in countries such as Italy (46%), Portugal (0.7%), Romania (2.1%), and Serbia (18.2%) [35,40,41,42]. In Asia, *E. canis* has been reported in dogs in India (16.1%), Korea (4.7%), and Pakistan (24.5%) [43,44,45]. In China, the prevalence of *E. canis* is 1.3% in dogs and 10.2% in ticks in southeastern regions [46]. In Xinjiang, *E. canis* is common, with a prevalence of 12.12% in dogs and 15.23% in ticks [47]. In Hong Kong, *E. canis* has been detected in stray dogs (8%) and pet dogs (6%) [48]. Similarly, in another study of 1508 dogs examined with real-time PCR, 7.4% were positive for *E. canis* [49]. 

Haemotropic mycoplasmas are small, unculturable, and cell wall-deficient bacteria that cause erythrocytic infections in domestic and wild mammals. *Mycoplasma haemocanis* and “*Candidatus* Mycoplasma haematoparvum” are two major species of hemoplasmas infecting dogs. Infections with canine hemoplasma are often asymptomatic, and the outcomes, including acute hemolytic anemia and fatal disease, may be associated with immunosuppression or coinfection with other pathogens. Canine hemoplasmas may be transmitted by bloodsucking arthropods. Particularly, *R. sanguineus* may play an important role as a vector in the transmission of these pathogens. In cats, blood transfusions are a common source of feline hemoplasma infections [50]. Because blood smears stained with Giemsa have low sensitivity and specificity, specific conventional or quantitative real-time PCR is used to examine the canine hemoplasma species [51]. In Europe, canine hemoplasma infections have shown a higher prevalence in countries with Mediterranean climate than in Switzerland [52,53]. *M. haemocanis* (8.45%) and *C.* M. haematoparvum (2.11%) have been detected in dogs in Portugal [35], and similar rates of *M. haemocanis* (9.9%) and *C.* M. haematoparvum (2.9%) have been detected in Cambodia [54]. In another study, the prevalence of *M. haemocanis* (26.2%) was higher than that of *C.* M. haematoparvum (6.7%) in Turkey [55]. *M. haemocanis* (38.2%) and *C.* M. haematoparvum (43.2%) are commonly found in Korea [44]. In Italy, the prevalence of *M. haemocanis* (13.1%) is similar to that of *C.* M. haematoparvum (11.4%) in hunting dogs [56]. The prevalence of canine hemoplasmas is low in Australia (1.6%) [57]. Coinfection with both hemoplasma species is found in dogs in Turkey (5.3%) and Italy (4.6%) [55,56]. In China, the prevalence of *M. haemocanis* is 1.85% in Jiangxi [58]. “*Candidatus* Mycoplasma haemominutum”, a major feline hemoplasma species, has been detected in China and Japan [59,60]. “*Candidatus* Mycoplasma haemobos” has also been reported in dogs in China [61].

Our understanding of the canine vector-borne pathogens in China remains scant. Therefore, in this study, we investigated the distribution and genetic characteristics of *Ehrlichia* spp., *Hepatozoon* spp., and *Mycoplasma* spp. in domestic dogs from Chongqing municipality and Fujian, Hubei, and Shandong provinces to update the current epidemic status of canine vector-borne diseases in China and to provide valuable reference information for the prevention and control of these diseases.

## 2. Materials and Methods

### 2.1. Sample Collection and DNA Extraction

Blood samples from 306 owned dogs were taken from pet clinics in the southwestern (Chongqing municipality), central (Hubei Province), and southeastern (Fujian and Shandong Provinces) regions of China. These regions have different geographical and environmental characteristics, such as temperature, humidity, and annual rainfall. The sample size was 203 dogs in Chongqing, 23 dogs in Fujian, 57 dogs in Hubei, and 23 dogs in Shandong. All samples were randomly collected from apparently healthy dogs, and no ectoparasites were found. Approximately 300 µL of whole blood were obtained in sterile EDTA vacutainer tubes and transported in iceboxes to the laboratory. Genomic DNA was extracted from 250 µL of blood using the Blood DNA Mini Kit (Omega, Norcross, GA, USA) following the manufacturer′s instructions and then stored at −20 °C until use. 

### 2.2. PCR Amplification and Sequencing

The presence of *Ehrlichia*, *Hepatozoon*, and *Mycoplasma* DNA was screened by conventional PCR. The primers EHR16SD: 5′-GGTACCYACAGAAGAAGTCC-3′ and EHR16SR: 5′-TAGCACTCATCGTTTACAGC-3′ were used to amplify the 345 bp fragment of the 16S rRNA gene for detection of *Ehrlichia* spp. [62]. A fragment of 666 bp of the 18S rRNA gene of *Hepatozoon* spp. was amplified using the primers: Hep F: 5′-ATACATGAGCAAAATCTCAAC-3′ and Hep R: 5′-CTTATTATTCCATGCTGCAG-3′ [63]. A PCR targeting approximately 560 bp of the 16S rRNA gene was performed using primers: Myco322s: 5′-GCCCATATTCCTACGGGAAGCAGCAGT-3′ and Myco938as: 5′-CTCCACCACTTG TTCAGGTCCCCGTC-3′ [64]. PCR reaction mixtures of 25 µl were prepared containing 2.5 µL of 10 × PCR buffer, 2.0 µL of 2.5 mM dNTP, 0.3 µL of 5 U/µL *Taq* DNA polymerase (Takara, Dalian, China), 0.1 µM of each primer, and 2 µL of DNA template under the reaction conditions as described previously with an annealing temperature of 53 °C for *Ehrlichia*, 58 °C for *Hepatozoon*, and 68 °C for *Mycoplasma* species [62,63,64]. The positive products were purified using a Hipure Gel Pure DNA Mini Kit (Magen, Guangzhou, China) and cloned into the pMD19-T vector (TaKaRa, Dalian, China). Sequencing reactions were performed with M13-F/M13-R primers, and the reaction products were separated and detected using an automated sequencer, the ABI 3730XL. DNA extracted from a dog infected with *H*. *canis* and distilled water were used as positive and negative controls, respectively.

### 2.3. Sequence Analysis

The 18S rRNA sequences obtained in this study were assembled and edited with the Lasergene program (DNASTAR Inc., Madison, WI, USA). To identify highly similar sequences, all these sequences were analyzed with the NCBI BLASTn program (https://blast.ncbi.nlm.nih.gov, accessed on 25 January 2022 and 20 June 2022). The accession numbers for the *Hepatozoon* isolates detected are OM392073–OM392078 and ON810476–ON810479.

ClustalW in the MEGA 11 software was used to align the sequences obtained [65]. A nucleotide sequence analysis was performed with the GeneDoc program [66]. To estimate the genetic relationships between different regions among *H*. *canis* isolates, a haplotype TCS network was constructed with the PopArt software [67,68]. The number of haplotypes, haplotype diversity, and nucleotide diversity were calculated with DnaSP 5.1 [69]. To infer the evolutionary relationships of the *H*. *canis* isolates, the sequences obtained in this study were compared with those from different countries and hosts downloaded from the GenBank database. A phylogenetic analysis with the neighbor-joining method was implemented in the MEGA 11 software [65]. The best Tamura 3-parameter model was selected. Branch support was assessed with bootstrap values calculated with 1000 replicates. A homologous sequence of *Adelina bambarooniae* (accession number AF494058) was used as an outgroup.

## 3. Results

### 3.1. Detection and Identification of Vector-Borne Pathogens

In the 306 samples analyzed, the prevalence of *Hepatozoon* spp. was 3.27%, but no *Ehrlichia* spp. or *Mycoplasma* spp. were detected, as shown in Table 1. *Hepatozoon* spp. were found in Chongqing and Hubei, with prevalence rates of 2.46% and 8.77%, respectively. BLAST analysis indicated that the 10 positive samples were closely related to *H*. *canis* from dogs in China (MT107091), Malaysia (KT267958), Zambia (LC331053), and Venezuela (DQ439540) with 96.3% to 100% sequence identity. The sequences obtained in this study shared 96.1% to 100% nucleotide identity with each other. 

### 3.2. Sequence Analysis

Nucleotide sequence variations were observed within the 18S rRNA sequences determined. Compared with the reference sequence (MT107091), nucleotide substitutions occurred at 34 positions, as shown in Figure S1. A haplotype analysis identified eight haplotypes in 10 individuals. The haplotype diversity and nucleotide diversity were 0.956 and 0.013, respectively.

### 3.3. Phylogenetic Analysis and Haplotype Network

**Figure 1:**
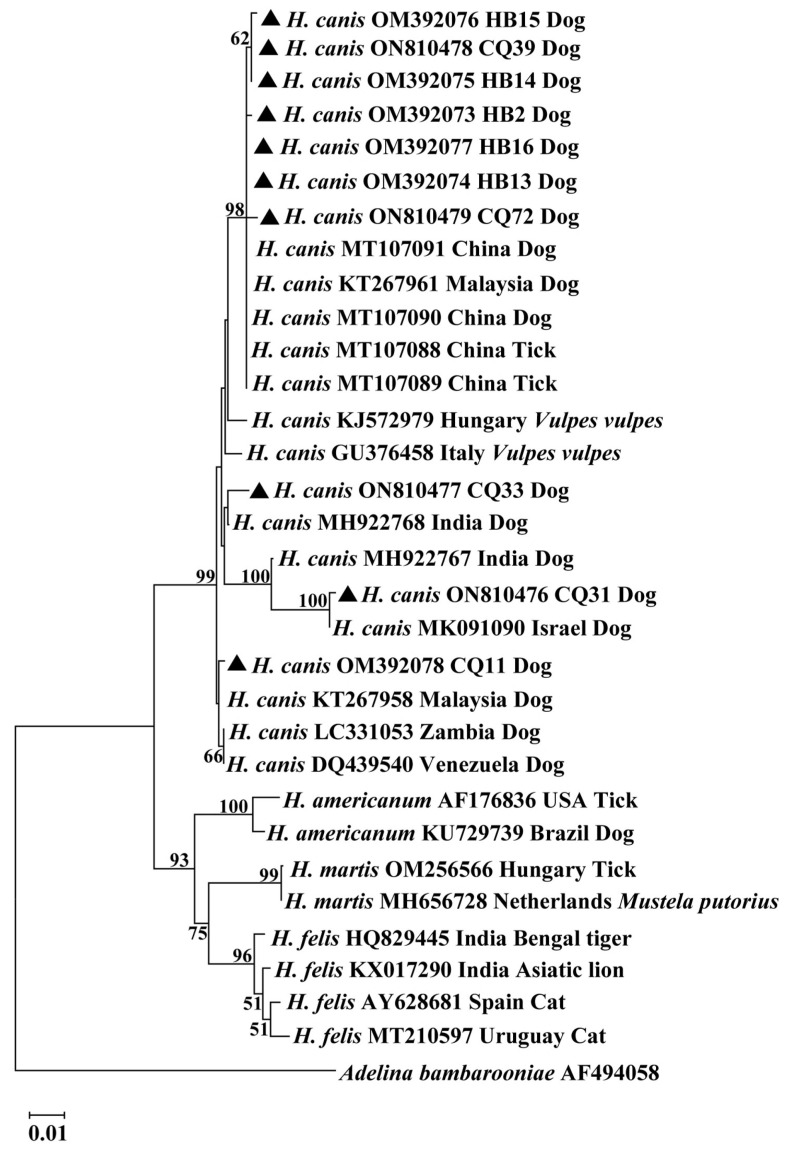
Phylogenetic tree constructed using 18S rRNA sequences from *Hepatozoon* isolates based on the neighbor-joining method. The sequences obtained from this study were compared with related sequences deposited in GenBank. The bootstrap values of >50% were exhibited at each branch point. GenBank accession numbers, the isolate, countries, and host were shown alongside species names. The sequence of *Adelina bambarooniae* (AF494058) was used as an outgroup. Representative isolates in this study were indicated by bold triangles. Abbreviations: CQ (Chongqing) and HB (Hubei).

A phylogenetic tree was constructed from the 18S rRNA sequences of the *H. canis* isolates together with related homologous sequences retrieved from GenBank, as shown in Figure 1. The neighbor-joining tree showed that the 10 *H. canis* isolates clustered into four clades. Seven *H. canis* isolates (CQ39, CQ72, HB2, HB13, HB14, HB15, and HB16) belonged to clade 1, and CQ33, CQ31, and CQ11 to clades 2, 3, and 4, respectively. The phylogenetic tree also indicated that *H. canis* isolates from various geographic regions or different hosts clustered in a large group with a bootstrap value of 99% but were clearly separated from those of other *Hepatozoon* species. 

**Figure 2:**
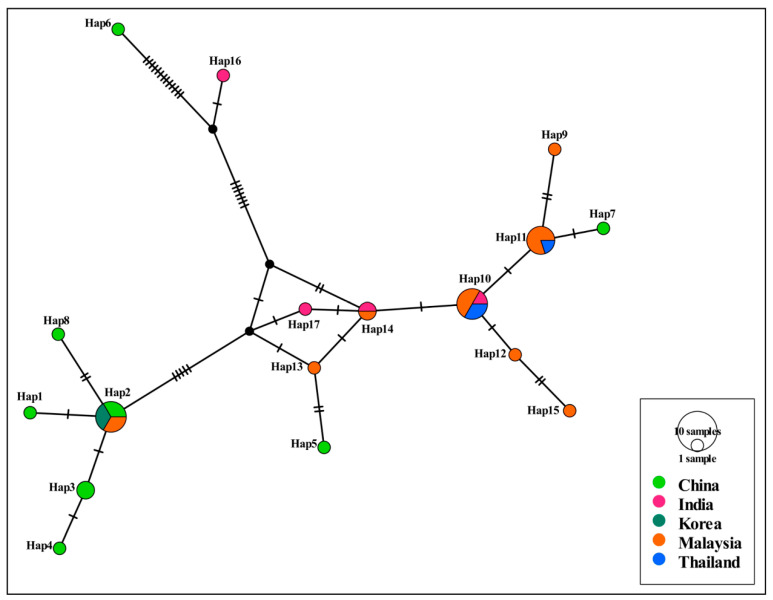
Haplotype network of *H. canis* isolates. The size of the circle represents the frequency of each haplotype. The different colored dots represent haplotypes from different locations.

The haplotype network showed that haplotypes Hap1, Hap2, and Hap4 originated in Hubei, haplotypes Hap5–Hap8 in Chongqing, and haplotype Hap3 in Chongqing and Hubei, as shown in Figure 2. Other haplotypes from India, Korea, Malaysia, and Thailand are shown in Figure 2. Haplotype Hap2 was shared by China, Korea, and Malaysia. The random distribution of eight haplotypes from China across the haplotype network did not display any particular genetic structure among *H. canis* isolates. The haplotype network also showed that there was no distinct grouping of *H. canis* isolates according to the geographical region in Asia.

## 4. Discussion

In China, pet ownership is rapidly growing in some cities. Keeping pets can not only enrich people’s spiritual lives, especially for empty-nesters, but also promote communication among pet owners and increase their happiness. An important problem should be noted: these pets may carry some pathogens, especially zoonotic pathogens. Thus, health issues for pet animals, their owners, or the public should not be neglected. In recent decades, the distribution and prevalence of CVBDs have been continuously expanding. This may be related to the interactions between pathogens, hosts, and vectors, which in turn, are influenced by environmental climate change and anthropogenic factors. In particular, arthropod vectors are more easily affected by climate change because high temperatures and low humidity contribute to their growth. Canine vector-borne pathogens are transmitted by arthropods, and many are zoonotic and threaten the health of animals and humans. Importantly, domestic dogs can act as reservoir hosts for zoonotic agents, and may transfer these pathogens to humans. 

In this study, the prevalence of common vector-borne pathogens was investigated in dogs in four regions of China. *H. canis* was identified in the collected samples by sequencing the 18S rRNA genes and was detected in 2.46% and 8.77% of the samples collected from Chongqing and Hubei, respectively. In previous studies, the prevalence of *H. canis* in dogs was 4.35% in Hanzhong [32], 4.5% in Beijing, 2.3% in Nanjing, 1.2% in Urumchi, 8.9% in Yangling, and 4.9% in Zhengzhou [31]. On a global scale, the prevalence of *H. canis* in China is lower than that in other countries such as Brazil (66.45%), Iran (23.07%), Pakistan (45.5%), and Portugal (20.42%) [35,70,71,72]. On the contrary, the prevalence of *H. canis* in some other countries, such as India (0.26%), Qatar (1.6%), and Thailand (1.81%), is lower than that in China [73,74,75]. The prevalence of *H. canis* in this study was also similar to that in guard dogs in Nigeria (6%) [76]. These differences in the distribution of *H. canis* could be related to differences in the abundance and geographical distributions of tick species, such as *Hae. longicornis* and *R. sanguineus*, differences in the characteristics of specific dog populations (e.g., age, sex, breed, and health), or the methods of sampling [77]. In China, more than 20 tick species have been recorded in Hubei, but relatively few in Chongqing [78]. In the present study, the prevalence of *H. canis* was higher in Hubei, where *R. sanguineus* is not endemic but *Hae. longicornis* is both endemic and common. In another study, a low prevalence of *Hepatozoon* sp. was found in *Hae. longicornis* in northeastern China [79]. Therefore, the role of *Hae. longicornis* as a vector transmitting hepatozoonosis should be investigated in future studies. No *H. canis* was detected in Fujian or Shandong, where *R. sanguineus* is considered endemic [78]. A possible explanation was that the sampled dogs had little opportunity to contact ticks or that the sample size was too small to detect their prevalence. *H. canis* is also the most prevalent pathogen in wild canids and has been described in red foxes in Poland, Portugal, Spain, Italy, and Germany [22,23,24,25,27], in golden jackals in Israel and Hungary [28,29], and in maned wolves in Brazil [80]. *H. canis* infections in foxes and dogs have both been detected in Italy and Germany [25,26,27], suggesting that it is transferred from wild canids to domestic animals living in the same areas. Currently, there is little information on *H. canis* in wild canids in China, and the relationship between *H. canis* infections in wild and domestic animals should be investigated in follow-up studies.

The four regions surveyed were negative for *Ehrlichia* spp. and *Mycoplasma* spp. These bacteria can be zoonotic and potentially fatal, and they are known to be transmitted by ticks. Among the three *Ehrlichial* species that infect dogs, *E. canis*, *E. chaffeensis*, and *E. ewingii*, *E. canis* is most common. The clinical manifestations of *E. canis* infection are variable, depending on the virulence of the strain, the immune status of hosts, and coinfection with other pathogens. *E. canis* has been reported in dogs in India, Italy, Korea, Pakistan, Portugal, Romania, and Serbia [35,40,41,42,43,44,45]. The prevalence of *E. canis* is high in India (16.1%) and Italy (46%) [40,43], indicating that dogs living outdoors are more susceptible to contact ticks compared with pet dogs living indoors. In addition, *E. canis* has also been examined in wild canids, including red foxes (52%), and gray wolves (50%), in Italy [81]. In general, *R. sanguineus* is considered the main vector for CME transmission, but *Hae. longicornis* is a common tick species responsible for transmitting CME in East Asian countries [82]. In China, *E. canis* has been detected in dogs in areas such as Beijing, Jiangsu, Xinjiang, and Hong Kong [31,47,48] and has been detected in other hosts, including ticks, goats, and deer [46,83,84]. *E. canis* has been identified both in *R. sanguineus* and *Hae. longicornis* in China [46]. *M. haemocanis* and *C. M. haematoparvum* are also common in dogs and have been detected in Australia, Cambodia, Italy, Korea, Portugal, and Turkey [35,44,54,55,56,57]. For *M. haemocanis* and *C. M. haematoparvum*, the prevalence of dogs living outdoors is higher than that living indoors [44,56], which are similar to *E. canis* infections in dogs. In Europe, *R. sanguineus* is mainly found in areas with Mediterranean climate, and a higher prevalence of canine hemoplasma infection was observed in these regions [52,53]. *M. haemocanis, C.* M. haemominutum, and *C.* M. haemobos have been identified in dogs in China [58,59,61]. However, our knowledge of *Ehrlichia* and *Mycoplasma* infections in dogs is still relatively limited, and large-scale epidemiological research is required in endemic or non-endemic areas for ticks in further studies. In this study, the failure to detect these pathogens could be related to the fact that the dogs sampled were domestic dogs maintained in good health and living conditions. Although these dogs spent most of their time indoors and had limited opportunities to contact ticks, *H. canis* was still detected. As a tick-borne disease, the prevalence of hepatozoonosis could be associated with the ability of ticks to transmit the pathogen. However, no ticks were detected on the bodies of these pet dogs or in the environment in which they lived, so it was difficult to identify the origins of the initial infections. Therefore, how these dogs were exposed to *Hepatozoon* infection and the routes of transmission of *H. canis* should be investigated in further studies. 

A haplotype analysis indicated high haplotype diversity (0.956), consistent with the findings for *H. canis* isolates from different continents in a previous study [85]. A haplotype network showed that haplotype Hap2 was shared by China, Korea, and Malaysia, which could be attributable to its evolution from other haplotypes in China or its introduction from neighboring countries. A phylogenetic tree showed that the *H. canis* 18S rRNA sequences clustered into four clades. This finding was similar to reports from Pakistan and Germany, where *H. canis* isolates were also classified into different clusters [71,86]. These sequence data suggested that genetic variation existed within *H. canis* isolates. In a phylogenetic analysis, *H. canis* did not clearly cluster according to geographic region. This result was supported by a recent study that showed no phylogeographic grouping when *H. canis* populations were analyzed by continent [85]. Collectively, these results imply the presence of minor strain variations in *H. canis*, but there may be some gene flow between different geographic regions. Possible reasons for this are that the transmission intensity of *H. canis* and the dispersal of ticks are affected by human activities and that the spread of ticks and tick-borne pathogens is influenced by migratory birds [85,87]. Further studies that evaluate the genetic characteristics of *H. canis* in large-scale samples from China are essential.

## 5. Conclusions

In this study, *H. canis* was detected in Chongqing and Hubei. High haplotype diversity and the occurrence of genetic variation were observed among these *H. canis* isolates. These findings provided a foundation for studying epidemiology and genetic characteristics of *H. canis* in China and will also be useful in understanding the health status of companion animals and minimizing the risk of infection in animals and humans.

## Figures and Tables

**Table 1:** Geographical distribution and prevalence of *Hepatozoon canis* in dogs in four regions of China.

Locations	Longitude Latitude	No. of Samples	No. of Positive Samples	Prevalence (%, 95% CI)
Chongqing	105.17–110.11° E, 28.10–32.13° N	203	5	2.46% (0.80–5.65)
Hubei	108.21–116.07° E, 29.05–33.20° N	57	5	8.77% (2.91–19.30)
Fujian	115.50–120.40° E, 23.30–28.22° N	23	0	0
Shandong	114.19–122.43° E, 34.22–38.23° N	23	0	0
Total		306	10	3.27% (1.58–5.93)

## Data Availability

All data generated or analyzed during this study are included in this published article.

## Supplementary Materials

The following supporting information can be downloaded at: https://www.mdpi.com/article/10.3390/ani13111867/s1. Figure S1. Nucleotide sequence analysis of *H. canis* isolates in dogs. The sequences obtained from this study were compared with the related sequences deposited in GenBank in China (MT107091), Malaysia (KT267958), Zambia (LC331053), and Venezuela (DQ439540). The nucleotide substitutions occurred at 34 positions. Abbreviations: CQ (Chongqing) and HB (Hubei).
