# Supplementary material for: Molecular Survey and Genetic Characteristics of Vector-Borne Pathogens in Domestic Dogs from Four Regions of China
Source: Animals (Basel). 2023 Jun 3;13(11):1867. doi: 10.3390/ani13111867 (PMC10251890; doi:10.3390/ani13111867)
Supplement: Supplementary file 1 [file animals-13-01867-s001.zip › animals-2386370-supplementary.pdf]

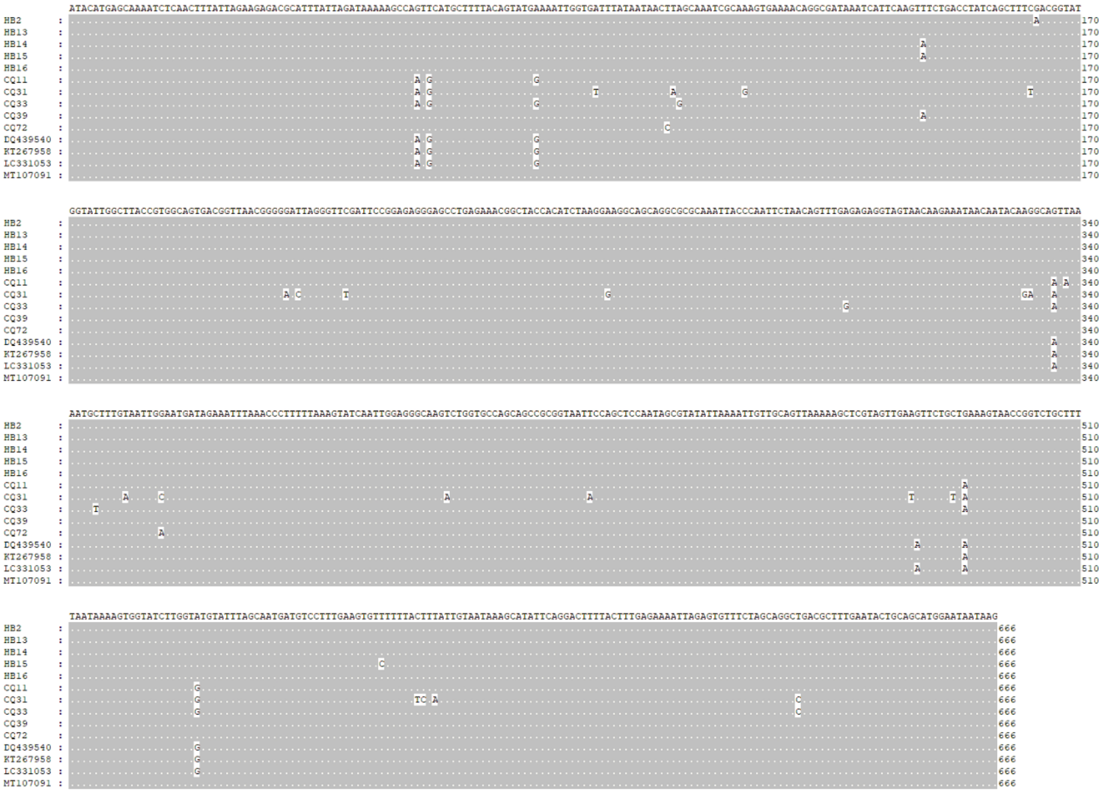

Figure S1. Nucleotide sequence analysis of *H. canis* isolates in dogs. The sequences obtained from this study were compared with the related sequences deposited in GenBank, China (MT107091), Malaysia (KT267958), Zambia (LC331053) and Venezuela (DQ439540). The nucleotide substitutions occurred at 34 positions. Abbreviations: CQ (Chongqing) and HB (Hubei).
